# Surface Protonics Promotes Catalysis

**DOI:** 10.1038/srep38007

**Published:** 2016-12-01

**Authors:** R. Manabe, S. Okada, R. Inagaki, K. Oshima, S. Ogo, Y. Sekine

**Affiliations:** 1 Applied Chemistry, Waseda University, 3-4-1, Okubo, Shinjuku, Tokyo, 169-8555, Japan; 2 Chemistry and Biochemistry, National Institute of Technology, Numazu College, 3600, Ooka, Numazu, Shizuoka, 410-8501, Japan

## Abstract

Catalytic steam reforming of methane for hydrogen production proceeds even at 473 K over 1 wt% Pd/CeO_2_ catalyst in an electric field, thanks to the surface protonics. Kinetic analyses demonstrated the synergetic effect between catalytic reaction and electric field, revealing strengthened water pressure dependence of the reaction rate when applying an electric field, with one-third the apparent activation energy at the lower reaction temperature range. *Operando*–IR measurements revealed that proton conduction via adsorbed water on the catalyst surface occurred during electric field application. Methane was activated by proton collision at the Pd–CeO_2_ interface, based on the inverse kinetic isotope effect. Proton conduction on the catalyst surface plays an important role in methane activation at low temperature. This report is the first describing promotion of the catalytic reaction by surface protonics.

Hydrogen has been used industrially for petroleum refining and to synthesize ammonia and methanol. Recently, hydrogen has attracted attention for wider use as a secondary energy source for hydrogen combustion or fuel cell utilization because hydrogen is converted easily into electric power, emitting only water from the reaction. Hydrogen demand is expected to increase along with the increasing use of fuel cell vehicles and home fuel cell systems. Catalytic steam reforming (SR) is now the most widely used method to produce hydrogen. The methane steam reforming reaction formula is presented below.


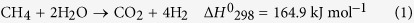


Generally, methane steam reforming is conducted at high temperatures (923–1123 K) with Ni catalysts^1^,^2^,^3^ because of thermodynamic equilibrium and because of methane molecule stability. However, such high reaction temperatures raise some issues such as the necessity for expensive materials that can tolerate high temperatures, catalyst deactivation, and complex processes with multiple heat-exchangers. Therefore, lowering the reaction temperatures can provide promising alternatives: low-grade waste heat might be reused effectively for hydrogen production because gaseous hydrogen itself has a high exergy ratio of 83%^4^,^5^. Lowering steam reforming temperatures not only has the potential to improve the hydrogen production efficiency, but also has high potential for waste heat recovery. This contribution can lead to more efficient heat and chemical energy conversion process.

Various methods have been investigated to lower reaction temperatures. Auto thermal reforming (ATR)^6^,^7^,^8^, which combines steam reforming and hydrocarbon combustion (using exothermic reactions), can operate at around 823 K. This process is simple, but nitrogen is included in its products, except when used with air separation. The process using non-equilibrium plasma^9^,^10^,^11^,^12^ showed high activity, even at ambient temperature, but the process consumes great amounts of electrical power. A non-faradaic reaction^13^ shows high activity with less electric power consumption because of the change of O^2−^ mobility and the work function of the catalyst surface. Especially NEMCA (non-faradaic electrochemical modification of catalytic activity) was investigated for use at temperatures of around 673 K^14^,^15^.

Our previous studies^16^,^17^,^18^ revealed that a steam reforming process in an electric field, *Electreforming* (ER), shows high activity even at a low temperature of 423 K. For ER, Pt or Pd catalysts supported on CeO_2_-based oxide are effective catalysts. Activities were increased drastically by the application of an electric field with lower electric power consumption of less than a few watts. We conducted kinetic measurements and *operando*-IR measurements for methane steam reforming in an electric field. Results show that the application of the electric field promoted surface protonics. Moreover, it enabled catalytic steam reforming even at the low temperature of 423 K.

## Kinetic analyses for catalytic steam reforming (SR) and *Electreforming* (ER)

**Figure 1:**
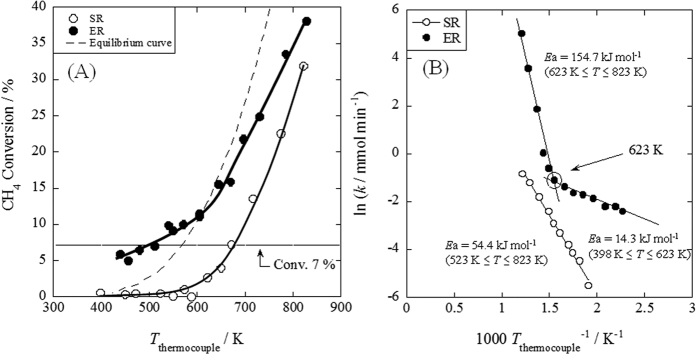
Temperature dependencies of catalytic activity with or without electric field. (**A**) Activities for steam reforming (SR) and *Electreforming* (ER), (**B**) Arrhenius plots for both reactions, preset temperature, 398–823 K; catalyst, 1.0 wt% Pd/CeO_2_, 80 mg; flow, CH_4_: H_2_O: Ar:He = 12: 24: 12: 72, total flow rate 120 SCCM; current, 0 or 5 mA.

Pd-catalyst supported on CeO_2_ showed high and stable activity for ER^17^. As described herein, we selected Pd/CeO_2_ as a catalyst for ER. Kinetic measurements were conducted over this catalyst to ascertain details of the effects of an electric field on catalytic methane steam reforming. The respective temperature dependencies for both reactions (SR and ER) are presented in Fig 1. These results were taken at a steady flow point within 5 min after applying the electric field, and the activity was stable over 6 h over Pd/CeO_2_ catalyst. As Fig. 1(A) shows, methane conversion increased drastically because of the application of an electric field to the catalyst bed, even at low temperatures. The activities for ER at low temperatures were over the limitation of thermodynamic equilibrium curve, indicating that the reaction in an electric field includes some irreversible elementary steps. Furthermore, results show that the methane conversions were almost identical (7%) for SR at 673 K and ER for 473 K. At around these temperatures, we also conducted measurements for partial pressure dependencies of both reaction rates. These results are summarized in Supplementary Materials Table S1. Regarding the reaction mechanism of conventional methane-SR, numerous investigations have been conducted^19^,^20^,^21^,^22^,^23^,^24^,^25^,^26^, revealing that the order for methane partial pressure (*α*) is almost 1; that for water pressure (*β*) is 0. Furthermore, the rate-determining step of methane steam reforming is the methane dissociative adsorption step (summarized in Supplementary Materials Table S2). However, different trends were observed for ER. At all four measured temperatures (473, 623, 673, and 723 K), the water pressure dependence was greater than that of methane pressure for ER. Comparison under the same conversion conditions for SR at 673 K and ER at 473 K (around at 7%) showed that application of the electric field increased the water pressure dependence from 0.25 to 0.79. These results demonstrate that drastic enhancement of the catalytic activity is not attributable to Joule heating because the partial pressure dependency of the reaction rate was clearly different, although these two reactions exhibited equivalent methane conversion. Using these results, rate constants were calculated assuming equation (2) as the reaction rate equation. Apparent activation energies (*E*_a_) were calculated for each reaction (Detailed procedures are also explained in Supplementary Materials).


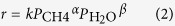


Arrhenius plots for both SR and ER are presented in Fig. 1(B), showing that the temperature dependency of reaction rates with and without the electric field differ considerably. The calculated apparent activation energy was 54.4 kJ mol^−1^ for SR. However, the slope of the Arrhenius plot for ER changed around 623 K. The apparent activation energy decreased by the application of electric field, especially at lower reaction temperatures of about 14.3 kJ mol^−1^. It was inferred from these results that the reaction mechanism of SR and ER differ markedly, and that ER proceeds with another reaction path having lower activation energy at temperatures lower than 623 K.

### *Operando* – diffuse reflectance infrared Fourier transform spectroscopy (DRIFTS) measurements – Qualitative changes for adsorbed species with/without electric field

**Figure 2:**
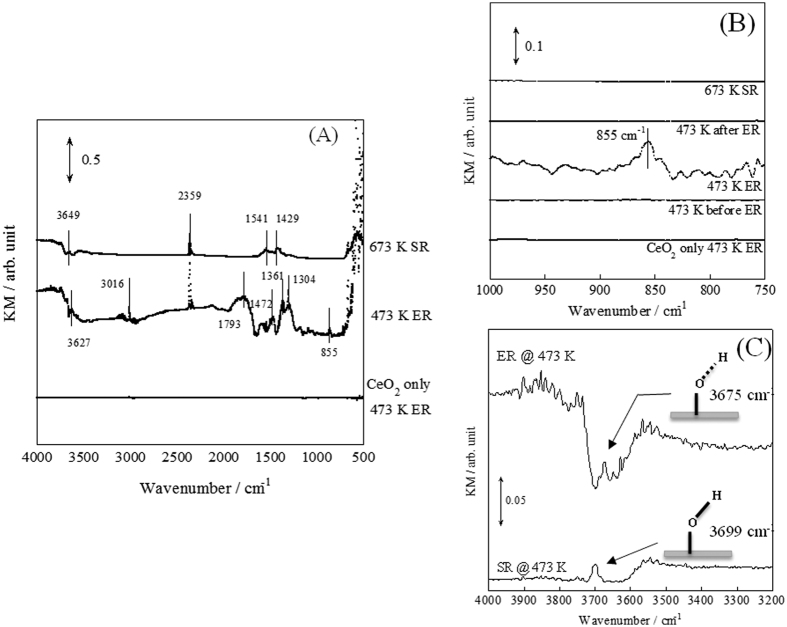
*Operando-*DRIFTS spectra with/without electric field. (**A**) Comparison for with/without Pd or application of electric field, (**B**) O-H rotating region, (**C**) O-H stretching region, catalyst, CeO_2_ or 1.0 wt% Pd/CeO_2_; flow, CH_4_: H_2_O: Ar = 1: 2: 62, total flow rate 65 SCCM; current, 0 or 5 mA.

Molecule adsorption on the catalyst surface is known to be feasible at lower temperatures. Therefore, to assess kinetic changes by the application of an electric field from the viewpoint of surface adsorbates, we conducted additional investigations using *operando*-diffuse reflectance infrared Fourier transform spectroscopy (DRIFTS) measurements. We measured DRIFTS spectra when an electric field was applied to the catalyst bed with a hand-made cell, as shown in Supplementary Materials Figure S2. The obtained *operando*-DRIFTS spectra are presented in Fig. 2. Assignments are presented in Supplementary Materials Table S5. For the spectra with the electric field (bottom and middle spectra in Fig. 2(A)), gas phase was removed by subtraction after the ER spectrum, which was recorded after ceasing application of the electric field. We can consider the role of Pd comparing the bottom and middle spectra, as presented in Fig. 2(A). The spectrum at 473 K ER using Pd-catalyst showed many adsorbed species. However, the spectrum at 473 K ER using CeO_2_ catalyst showed no adsorbates, indicating that no adsorption enhancement proceeded by application of electric field without supported Pd. Therefore, Pd supported on CeO_2_ is regarded as an active site for ER. Additionally, we can compare the observed adsorbates with or without an electric field, using middle and top spectra, as presented in Fig. 2(A). Clearly different spectra were obtained with and without the electric field. These two reactions, SR at 673 K and ER at 473 K, showed methane conversion that is the same as those presented in Fig. 1(A). This result gives evidence that reaction mechanisms with and without an electric field differ markedly. The result also demonstrates that Joule heat is unimportant for ER, as suggested by kinetic analysis results.

The spectra at 473 K ER provide more detailed information related to the qualitative change for adsorbed water. As shown in Fig. 2(B), the peak assigned to rotation of adsorbed water^27^ (855 cm^−1^) was observed only when an electric field was applied to Pd-catalyst with methane and water at a low temperature: 473 K. This peak is regarded as having a strong relation with the Grotthuss mechanism, a widely known mechanism of proton conduction. By this mechanism, protons hop via water molecules with a distorted O–H bond. The rate-determining step of the Grotthuss mechanism is reported as the water rotation step^27^,^28^,^29^. The distorted O–H bond species were also observed during ER, as shown in Fig. 2(A) and (C). When applying an electric field, the peak assigned to O–H stretching^30^ shifted to a lower wavenumber. This red-shift of O–H stretching peak was greater than 20 cm^−1^, corresponding to the weakness of O–H bond energy about 25 kJ mol^−1^ (also see Supplementary Materials). Therefore, activated water exists on the catalyst surface, related to proton hopping. CeO_2_ itself also shows the electrical property of proton conduction by virtue of adsorbed water on its surface, as evaluated using our AC impedance measurements (described in Supplementary Materials). These results reveal that proton hopping occurred via adsorbed water on the catalyst surface during ER.

### *Operando*-DRIFTS measurements – Quantitative changes for adsorbed species with/without electric field

**Figure 3:**
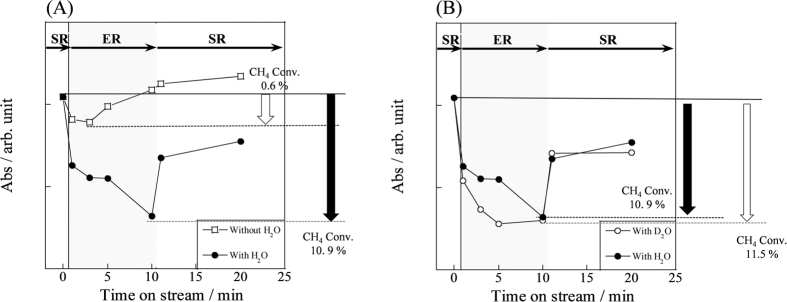
IR band intensities of CH_4_ gas (3016.6 cm^−1^) at 473 K in electric field. (**A**) With/without H_2_O, (**B**) with H_2_O/D_2_O, catalyst, 1.0 wt% Pd/CeO_2_; flow, CH_4_: H_2_O/D_2_O: Ar = 1: 2(0): 62(64), total 65 SCCM; current, 5 mA.

To elucidate the relation between proton conduction and catalytic activity enhancement by the application of an electric field, we specifically addressed the quantitative change for gas phase methane. Figure 3 presents IR band intensities derived from gas phase methane^31^ at 3016.6 cm^−1^ with or without an electric field under various conditions. As shown in Fig. 3(A), the gas-phase methane intensity decreased drastically, resulting in adsorption by the application of the electric field under the water-supplied condition. However, its intensity was almost equal with or without an electric field under conditions in which only methane is supplied. Indeed, methane was converted only slightly into CO and CO_2_ without water. However, methane conversion with water exceeded 10%, by *operando*-analyses, as presented in Table S6. These results demonstrate that the process of methane activation at low temperature has a relation with water, i.e., with proton hopping at the catalyst surface. Assuming that methane is activated by proton collision derived from the Grotthuss mechanism, we also conducted detailed investigations using D_2_O from the viewpoint of an inverse kinetic isotope effect (inverse KIE, also explained in Supplementary Materials). Inverse KIE theory^32^,^33^,^34^,^35^ revealed that the vibration mode, which has stronger C–H–H coupling bond than that of C–H–D, increases the difference of energy level between the transition state and dissociative chemisorption state. Therefore, the rate constant calculated when using isotope (*k*_D_) becomes larger than that when the isotope is not used (*k*_H_). Our analyses conducted using *operando*-DRIFTS showed good agreement with this theory. As shown in Fig. 3(B), the amount of gas phase methane decreased more when D_2_O was used instead of H_2_O. The decreased methane was then converted into CO and CO_2_, revealed by our *operando* analyses. These results support the inference that the methane activation process includes proton hopping on the catalyst surface.

**Figure 4:**
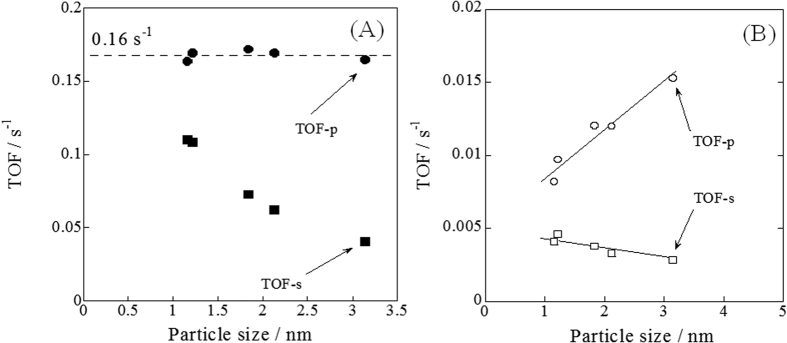
Pd specific surface area (TOF-s) dependency or Pd perimeter (TOF-p) dependency of activities for ER at 473 K and SR at 673 K. (**A**) ER at 473 K, (**B**) SR at 673 K, catalyst, 1.0 wt% Pd/CeO_2_; flow, CH_4_: H_2_O: Ar = 1: 2: 62, total 65 SCCM; current, 0 or 5 mA.

Generally, water is regarded as adsorbed onto the catalyst support (CeO_2_), rather than loaded metal (Pd). For that reason, methane activation with proton is likely to occur at the Pd–CeO_2_ interface. To elucidate the reaction site for both SR and ER, we evaluated the turnover frequency with various amounts of Pd-loaded catalyst. Figure 4 presents the Pd specific surface area dependency or Pd perimeter dependency of activities for ER at 473 K and SR at 673 K, as analyzed using *operando*-DRIFTS and CO pulse. We consider two kinds of turnover frequency (TOF), as determined by the Pd-specific surface area (TOF-s) and Pd perimeter (TOF-p). Detailed procedures for calculations are presented in Supplementary Materials. Generally, the methane steam reforming activity is determined by the active site of the loaded metal surface area^21^. Regarding SR without the electric field, we obtained the same trends, as shown in Fig. 4(B). The SR activity showed dependence on the Pd-specific surface area, rather than the Pd perimeter. However, these trends differed drastically for ER with application of the electric field. The ER activity showed strong dependence on the Pd perimeter, rather than on the Pd specific surface area, which indicates that the ER reaction proceeds mainly at the Pd–CeO_2_ interface.

**Figure 5:**
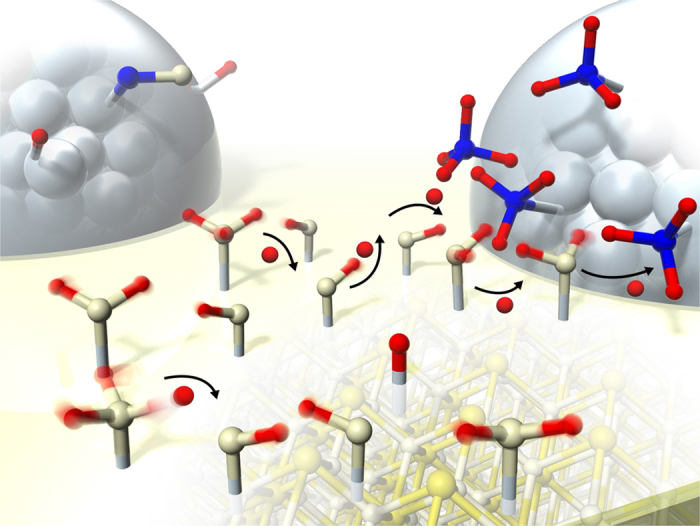
Schematic image of reaction mechanism for *Electreforming*.

Our *operando* analyses revealed that proton hopping promotes methane activation at the Pd–CeO_2_ interface. Furthermore, our *in-situ* XAFS measurements, presented in Supporting Information Figures S19 and S20, demonstrated that the electronic state (work function) and the structure of Pd/CeO_2_ catalyst were not changed by application of the electric field. Therefore, surface protonics is a dominant factor for the activity increase. A schematic image of the mechanism for *Electreforming is* presented in Fig. 5. Methane is activated at the interface by hopping proton derived from Grotthuss mechanism, resulting in adsorption onto Pd. By application of the electric field to methane steam reforming, methane can be activated at a low temperature, which never occurs without the electric field. Applying an electric field enables surface protonics via adsorbed water on the catalyst. It promotes a surface catalytic reaction.

## Conclusion

Methane steam reforming (SR) over 1 wt% Pd/CeO_2_ in the electric field, so-called *Electreforming* (ER), was performed. Application of an electric field increased the activity drastically. To elucidate the effects of an electric field on methane steam reforming, kinetic investigations and *operando*-DRIFTS measurements were conducted.

Results of our kinetic analyses demonstrated that the water pressure dependency of the reaction rate increased during the application of the electric field. However, the apparent activation energy decreased with an electric field, especially at lower reaction temperatures, indicating that the reaction mechanisms with and without electric field differ considerably.

Results of *operando*-DRIFTS revealed that proton conduction via adsorbed water on catalyst surface occurred with an electric field, known as the Grotthuss mechanism. Furthermore, our *operando* analyses from the viewpoint of an inverse kinetic isotope effect elucidated that methane was activated by proton collision derived from the Grotthuss mechanism. Furthermore, ER proceeds mainly at the interface between Pd and CeO_2_. Therefore, the surface protonics by the application of electric field serve an important role in the enhancement of catalytic methane steam reforming at a low reaction temperature.

## Methods

### Preparation of catalysts

For this study, CeO_2_ (JRC-CEO-1) was used as the catalyst support. Using CeO_2_, Pd supported catalyst (Pd/CeO_2_) was prepared with an impregnation method. Pd(OCOCH_3_) (Kanto Chemical Co. Inc.) was used as a metal precursor. First the distilled water solvent with CeO_2_ was evaporated at room temperature *in vacuo* for 2 h. Then the solvent with Pd precursor was added and stirred for another 2 h. The obtained solution was heated and stirred at 343 K. Then it was dried at 393 K for 20 h. The dried sample was heated in an oven at 973 K for 12 h. The respective Pd contents were 0.5, 1, 2, 3, and 5 wt%. The prepared catalyst was crushed to particles of 355–500 μm.

### Catalytic activity tests

For all activity tests, a quartz tube (6.0 mm i.d.) was used as a fixed-bed flow-type reactor. Two stainless steel rods (2 mm o.d.) were inserted into the reactor as electrodes. The upper electrode was set on the top of the catalyst bed. The ground electrode was set on the bottom of the catalyst bed. The electrode gap distance was fixed at 1.1 mm. A schematic image of the reactor is portrayed in Supplementary Materials Figure S1. The catalyst bed temperature was measured using a thermocouple. The imposed current and response voltage waves were observed using a digital phosphor oscilloscope (DPO4034B; Tektronix Inc.). Activity tests were conducted fundamentally using 80 mg catalyst (catalyst height: 1.6 mm) under CH_4_ (12 SCCM), H_2_O (24 SCCM), Ar (as internal standard gas, 12 SCCM), and He (as balance gas, 72 SCCM) flow at various preset temperatures, with 5 mA (DC). The total flow rate of the supply gas was 120 SCCM. Product gases were analyzed qualitatively and quantitatively using a Q-Mass (QGA; Hiden Analytical Ltd.). Methane conversion was calculated using the ratio of output moles of carbon atom in product species (CO and CO_2_) and input moles of carbon atom in methane. The reaction rate (*r*) corresponds to the sum of the produced CO flow rate and the CO_2_ flow rate.

### *Operando*-DRIFTS measurements

To elucidate the adsorbed species on catalysts with or without an electric field, *operando*-DRIFTS measurements were conducted using FT-IR (FT-IR6200; Jasco Corp.) with an MCT detector and a diffuse reflectance infrared Fourier transform spectroscopy reactor cell (DR-600Ai and DR-600Ci; Jasco Corp.) with a ZnSe window. For IR measurements with application of the electric field, DRIFTS cells made of SUS304 for catalytic reaction (for tolerance of high temperatures) and of Teflon for catalytic reaction in the electric field (to avoid short circuits in the cell) were used, as presented in Supplementary Materials Fig. S2. The sample was x wt% Pd/CeO_2_ (0 ≤ x ≤ 5). First, background (BKG) measurements were taken under inert Ar gas (62 SCCM) at 473 or 673 K. Subsequently, the reactant gases (CH_4_: H_2_O or D_2_O: Ar = 1: 2: 62, total 65 SCCM) were supplied for about 30 min at 473 K (or 673 K for SR, and temperature was down to 473 K with only Ar). Then the electric field was applied for about 10 min. Each spectrum was recorded at resolution of 2.0 or 4.0 cm^−1^ over 10 or 50 scans. The imposed current was 5 mA. The spectrum after ER was subtracted from the ER spectrum to remove the gas phase component in cases of necessity. The activities were evaluated using GC-FID (GC-2014; Shimadzu Corp.) for *operando* analyses.

### Characterization of catalyst

The dispersion ratio and particle diameter of Pd catalysts were characterized using CO pulse (BEL CAT II; Microtrac-Bel Japan Inc.). Before measurements, the catalyst sample was pre-treated under He flow at 473 K for 1 h. After the treatment, the temperature was decreased to 323 K with He, and 10% CO was pulsed. The results of CO pulse are presented in Supplementary Materials Table S9. TOF-s and TOF-p were calculated with the number of surface Pd atoms and that of interfacial Pd atoms. Detailed procedures are presented in Supplementary Materials. Pd and Ce *K*-edge *in-situ* X-ray adsorption fine structure (*in-situ* XAFS) spectra were recorded on BL14B2 in SPring-8 (Hyogo, Japan). The 3 wt% Pd/CeO_2_ catalyst was pressed into a pellet. Then the pellet was attached to a cell prepared for *in-situ* measurements, as shown in Figure S18. The pellet sample was pre-treated with Ar flow at 723 K for 30 min. Measurements were conducted with 5 mA current and CH_4_: H_2_O: Ar = 1: 2: 117, total 120 SCCM flow at 473 K. Software (Athena ver. 0.8.056, Artemis ver. 0.8.012) was used to analyze the obtained XAFS spectra. Phase and morphology of CeO_2_ disc for AC impedance measurements were analyzed by X-ray diffraction (XRD, MiniFlex 600, Cu *K*_α1_, Rigaku) and scanning electron microscopy (FE-SEM, Quanta 200 FEG, FEI Company).

### AC Impedance measurements

Alternating current (AC) impedance measurements were taken to evaluate the electrical properties of CeO_2_. For the measurements, we prepared a disc-shaped CeO_2_ sample. First, CeO_2_ powder was suspended in *iso*-propanol and was crushed into fine particles using a ball mill (rotation at 300 rpm for 15 min, then pause for 10 min, and rotation again at 300 rpm for 15 min; Fritsch GmbH). After evaporation of *iso*-propanol, the powder was pressed at 2 ton for 2 min to produce a disc. The disc was calcined in air at 1273 K for 2 h. For attachment of electrodes to the disc, Pt ink (Metalor UK, Pt ink number 6926) was painted on both sides of the disc, which was then calcined at 1173 K for 1 h. The diameter of the electrodes was 7.83 mm, and the thickness of the disc was 0.97 mm. These geometric factors were used for calculating conductivities. Electrical conductivities were measured using a ProboStat measurement cell (NorECs, Norway) with a standard 2-electrode-4-wire setup and connected to a Novocontrol alpha-A impedance spectrometer with a ZG4 interface, in dry Ar and wet Ar flow (*P*_H2O_ = 0.026 atm) at 398–673 K for wet and 423–773 K for dry condition. The impedance spectroscopy were measured within the frequency range 10^−3^ to 10^7^ Hz, with an oscillation voltage of 0.1 V RMS. Data were analyzed using ZVIEW equivalent circuit fitting software (version 3.5a, Scribner Associate Inc.). The models of equivalent circuit and the results are shown in Supplementary Materials Figure S16.

## Additional Information

**How to cite this article**: Manabe, R. *et al*. Surface Protonics Promotes Catalysis. *Sci. Rep.* **6**, 38007; doi: 10.1038/srep38007 (2016).

**Publisher's note:** Springer Nature remains neutral with regard to jurisdictional claims in published maps and institutional affiliations.

## Supplementary Material

Supplementary Information
