# Supplementary material for: Surface Protonics Promotes Catalysis
Source: Sci Rep. 2016 Dec 1;6:38007. doi: 10.1038/srep38007 (PMC5131306; doi:10.1038/srep38007)
Supplement: Supplementary Information [file srep38007-s1.pdf]

Title

Surface Protonics Promotes Catalysis

Authors

R. Manabe[1], S. Okada[1], R. Inagaki[1], K. Oshima[2], S. Ogo[1], Y. Sekine[1]*

Affiliations:

[1]Applied Chemistry, Waseda University, 3-4-1, Okubo, Shinjuku, Tokyo, 169-8555 Japan

[2]Chemistry and Biochemistry, National Institute of Technology, Numazu College, 3600, Ooka, Numazu, Shizuoka, 410-8501 Japan

*Corresponding author: Yasushi Sekine, ysekine@waseda.jp

Supplementary Materials:

Apparatus and IR cell for electric field application to a catalyst bed

We prepared the original reactor for application of the electric field to the catalyst bed. A schematic image of the reactor is portrayed in Figure S1. In addition, a hand-made Teflon cell was used for conducting *operando*-DRIFTS measurements while an electric field was applied to the catalyst bed. A schematic image of Teflon cell for *operando*-DRIFTS measurements is presented in Figure S2.

Kinetics of Methane Steam Reforming (SR)

Numerous investigations for conventional methane steam reforming (SR) have been conducted, revealing strong dependence of methane pressure on the reaction rate over various catalysts observed at 823–1073 K, except for Pd/CeO_2 catalyst at 620–770 K (*19–26*), as presented in Table S2. Reportedly, the rate-determining step of methane steam reforming is the methane dissociation step, derived from a stable C–H bond. Our results of kinetic analyses over Pd/CeO_2 catalyst, without an electric field, showed that the dependences of methane pressure and water pressure on the reaction

rate were almost identical, about 0.25, around 623–723 K, as shown in Table S1. These experiments were conducted under 80 mg weight of catalyst and 120 SCCM total flow rate, to arrange the same conditions for *Electreforming* (ER). Therefore, we conducted other experiments under the same conditions as those used with previous work (*19–23*,*25*): 5 mg weight of Pd/CeO_2 catalyst + 500 mg weight of SiO_2, and 180 SCCM total flow rate at 823 K, with a smaller amount of catalyst. The methane and water pressure dependence of the reaction are presented in Figure S3. We obtained the same trends as those reported from earlier studies, showing that the order for methane partial pressure is almost 1 and that for water is 0. From these results, we inferred that the mechanism of SR with our prepared Pd catalyst showed the same mechanism as that reported, indicating that the rate-determining step is the methane dissociative adsorption step.

Calculation of rate constant *k* for SR and ER

To evaluate the apparent activation energy (E_a), we calculated rate constant (k). The reaction rate equations are estimated as equations (1)–(3) from our kinetic analyses, as shown in Tables S1 and Table S3. For SR, we ascertained the reaction rate equation as equation (2) because our measurements for the dependence of partial pressure were almost identical around 623–723 K. The orders for methane and water pressure were average values. However, for ER, we extracted the increased reaction rate (r_{ER}) with equation (1). The apparent activation energy was calculated using k_{ER}.

$$r = k\, P_{CH4}^{\alpha}\, P_{H2O}^{\beta} = r_{SR} + r_{ER} \qquad (1)$$

$$r_{SR} = k_{SR}\, P_{CH4}^{0.27} + P_{H2O}^{0.26} \qquad (2)$$

$$r_{ER} = k_{ER}\, P_{H2O}^{\beta'} \qquad (3)$$

Our kinetic analyses for r_{ER} revealed that the methane pressure dependence of (α') r_{ER} was approximately 0, and that the water pressure dependence (β') changed with the reaction temperature, as presented in Figure S4. Therefore, we used β' at each reaction temperature from measured values

and estimated values. The estimated values were obtained as an average from measured values. The obtained orders for *β'* are presented in Table S4.

Operando-DRIFTS spectra and analyses for products via SR and ER under various conditions

Operando-DRIFTS spectra before subtracting after ER spectrum at 473 K with Pd/CeO_2 or CeO_2 catalyst are shown in Figure S5. The assignments are presented in Table S5. Furthermore, *operando*-DRIFTS spectra over Pd/CeO_2 catalyst with H_2O/D_2O or without water (only CH_4) are shown in Figure S6. The *operando*-DRIFTS spectra over Pd/CeO_2 catalyst with CH_4 and H_2O/D_2O at various temperature are presented in Figures S7 and S8. Results of analyses of products are presented in Tables S6 and S7.

Calculation of O–H bond energy from wavenumbers of IR spectrum

As shown in Figure 2(A) and Figure 2(C), the red-shift of O–H stretching peak was observed by the application of an electric field of 3699 cm^{-1} to 3675 cm^{-1} or from 3649 cm^{-1} to 3627 cm^{-1}. Using these wavenumbers and following equations (4) and (5), the difference of O–H bond energy was calculated.

$$v_{\text{O–H}} = \frac{1}{2\pi c}\sqrt{\frac{k}{\mu}} \qquad (4)$$

$$\Delta E = \frac{1}{2}\,\Delta k\,x^2 \qquad (5)$$

In those equations, *v* denotes the wavenumber of O–H peak from experimentally obtained results, *c* stands for the velocity of light, *k* signifies the O–H bond constant, *μ* denotes the reduced mass between O and H, *E* represents the O–H bond energy, and *x* is the O–H bond length of 1.02 Å (*36*). Our calculations show that ΔE is approximately 25 kJ mol^{-1}. The O–H bond energy was weakened about 25 kJ mol^{-1} by application of the electric field.

Inverse Kinetic Isotope Effect (Inverse *KIE*)

KIE is defined as the ratio of k_D/k_H, where k_D is the rate constant of the reaction with D_2O, and where k_H is the rate constant of the reaction with H_2O. With this conversion, KIEs greater than unity are called “inverse.” Those less than unity are called “normal” (*33*). As Figure 3 shows, the decrease of gas phase methane was greater with D_2O, than with H_2O. Assuming that k_D/k_H represents the ratio of the gas phase methane decrease, our results show that inverse KIE was observed around 473–573 K during ER, presented in Figure S9. These results strongly support our assumption that methane is activated by proton collision derived from the Grotthuss mechanism.

TOF determined by Pd specific surface area (TOF-s) and Pd perimeter (TOF-p)

To evaluate the influence of the number of Pd active site or the length of Pd interface on the activities for ER at 473 K and SR at 673 K, we prepared different amounts of Pd-loaded catalyst and conducted *operando*-DRIFTS measurements with this catalyst. *Operando*-DRIFTS spectra are presented in Figures S10 and S11, and the analyses for products are presented in Table S8. Using these results and the result of CO pulse, presented in Table S9, TOF-s and TOF-p were calculated. To evaluate the particle size of Pd on CeO_2 correctly, the influence of CO adsorption onto CeO_2 was examined first. Figure S12 shows the results of CO pulse for CeO_2 and 1.0wt%Pd/CeO_2 catalyst. As Figure S12 shows, the GC intensity of CO for CeO_2 at first time and the saturated GC intensity of CO for 1.0wt%Pd/CeO_2 were almost the same. Figure S13 shows IR spectra recorded 1 h after supplying nearly 40% CO flow with Ar at 323 K for CeO_2 and 1.0wt%Pd/CeO_2 catalyst. After purged with Ar for 1 h, the adsorbed CO remained only on Pd loaded catalyst. From these results, we concluded that the influence of CO adsorption onto CeO_2 was negligible when using CO pulse dosing.

As shown in Table S9, the particle size of Pd was almost the same for 0.5wt% and 1.0wt% Pd loaded catalysts, about 1.0 nm. And the particle size became larger than 1.0 nm for more than 1.0wt%

Pd loaded catalysts. For calculation of TOF-p, the Pd–Pd bond length was defined as 2.72 Å (*37*). The following equations (6) and (7) were used.

$$\text{TOF-s } [/s^{-1}] = \frac{Converted\ mole\ number\ of\ CH4}{Pd\ atom\ number\ at\ Surface} \quad (6)$$

$$\text{TOF-p } [/s^{-1}] = \frac{Converted\ mole\ number\ of\ CH4}{Pd\ atom\ number\ at\ Perimeter} \quad (7)$$

AC Impedance measurements for evaluating proton conduction via adsorbed water on surface of CeO_2

To evaluate the electrical properties of CeO_2, we conducted AC impedance measurements. The results of characterization (XRD and SEM) are shown in Figure S14. The phase of CeO_2 was cubic, and the average particle size of bulk was 110 nm. The relative density of CeO_2 disc was 61%, so it has a kind of pores, as shown in SEM images. Figure S15 presents the examples of Nyquist plots and fitting results. At temperatures from 423 to 673 K, all Nyquist plots obtained under wet condition (P_{H2O} = 0.026 atm) showed smaller arcs rather than those under dry condition. Therefore we considered the parallel equivalent circuit, as shown in Figure S16, where *R* stands for the resistance, CPE represents the capacitance with constant phase element, and $_{b,\ gb,\ surf.\ b}$ and $_{surf.gb}$ respectively correspond to a bulk, a grain boundary, a surface of bulk, and a surface of grain boundary. The disc of CeO_2 has some pores in itself, the surface conduction via adsorbed water on bulk and grain boundary could be observed. First, the data under dry condition were fitted, and we obtained *R*, *C* (Capacitance), and CPE values respectively. Then these parameters under dry condition were kept fixed, and the data under wet condition were fitted to obtain surface *R*, *C* and CPE values respectively. Also, we can exclude the component of electrodes with Nyquist plots and equivalent circuit in case of necessity.

Figure S17 shows the temperature dependency of conductivity under dry and wet conditions. The results under dry condition at high temperatures (573 < T < 773 K) showed the typical temperature

dependency of CeO_2 on conductivity with mixed (ionic and electronic) conduction (*38-40*). The apparent activation energies under high temperature region were 1.19 eV for grain boundary and 1.30 eV for bulk. However the apparent activation energies decreased to 0.36 eV for grain boundary and 0.27 eV for bulk respectively even under dry condition at lower temperatures ($423 < T < 573$ K). These results are considered to reflect the mobility of lattice oxygen. It is reported that the lattice oxygen of CeO_2 starts to move around over 600 K (*41*). Therefore, at low temperatures, the electron conduction is dominant and the barrier for electron hopping is considered to be relatively low (*40*). The results under wet condition ($\sigma_{surf.\ b}$ and $\sigma_{surf.gb}$) showed higher conductivity compared to those (σ_b and σ_{gb}) under dry. These two conductivity is considered to present proton conductivity, especially via adsorbed water onto the bulk and grain boundary surface, because $\sigma_{surf.\ b}$ and $\sigma_{surf.gb}$ became larger with lowering temperature at lower temperatures ($T < 573$ K for $\sigma_{surf.\ b}$ and $T < 448$ K for $\sigma_{surf.gb}$). These phenomena was studied, for example with TiO_2 (*42*), and the reason conductivity increases under lower temperatures is considered to be related with increasing the amount of adsorbed water on the surface of bulk and grain boundary according to Grotthuss mechanism: proton hopping. From these results, it is revealed that the surface protoics could be occurred via adsorbed water onto CeO_2.

.

In-situ XAFS measurements for evaluating the electronic state and structure of Pd/CeO_2 catalyst during ER

To evaluate the change of electronic state and structure for Pd/CeO_2 catalyst, we conducted *in-situ* XAFS measurements at SPring-8, Hyogo in Japan using an *in-situ* cell as shown in figure S18. Figures S19 and S20 show the obtained XAFS spectra with an electric field for the Pd-*K* edge and Ce-*K* edge at 473 K. As presented in Figure S19(a), Pd was slightly oxidized with the supply of raw materials: CH_4 and H_2O. Pd was then reduced with application of an electric field because the reaction proceeded and hydrogen was produced. However, no drastic change of the electronic state for Pd was confirmed,

suggesting that the main effect of the electric field is not the change for work function of loaded metal (Pd) on catalyst. The EXAFS spectra shown in Figure S19(b) show that the Pd structures with and without application of the electric field are almost identical. Additionally, as presented in Figures S20(a) and S20(b), neither the electronic state nor structure of CeO_2 was changed by application of the electric field. These results demonstrate that the electronic state and the structure of Pd/CeO_2 catalyst are almost identical, and that the electric field mainly promotes protonics on the catalytic surface via adsorbed water.

References (cited in this SI)

19. Wei, J., Iglesia, E., Isotopic and kinetic assessment of the mechanism of methane reforming and decomposition reactions on supported iridium catalysts. *Phys. Chem. Chem. Phys.*, **6**, 3754–3759, (2004).

20. Wei, J., Iglesia, E., Reaction pathways and site requirements for the activation and chemical conversion of methane on Ru-based catalysts. *J. Phys. Chem. B.*, **108**, 7253–7262 (2004).

21. Wei, J., Iglesia, E., Mechanism and site requirements for activation and chemical conversion of methane on supported Pt clusters and turnover rate comparisons among noble metals. *J. Phys. Chem. B.*, **108**, 4094–4103 (2004).

22. Wei, J., Iglesia, E., Structural requirements and reaction pathways in methane activation and chemical conversion catalyzed by rhodium. *J. Catal.*, **225**, 116–127 (2004).

23. Wei, J., Iglesia, E., Isotopic and kinetic assessment of the mechanism of reactions of CH_4 with CO_2 or H_2O to form synthesis gas and carbon on nickel catalysts. *J. Catal.*, **224**, 370–383 (2004).

24. Laosiripojana, N., Assabumrungrat, S., Methane steam reforming over Ni/Ce-ZrO_2 catalyst: Influences of Ce-ZrO_2 support on reactivity, resistance toward carbon formation, and intrinsic reaction kinetics. *Appl. Catal. A: Gen.*, **290**, 200–211 (2005).

25. Yamaguchi, A., Iglesia, E., Catalytic activation and reforming of methane on supported palladium clusters. *J. Catal.*, **274**, 52–63 (2010).

26. Wang, X., Gorte, R. J., A study of hydrocarbon fuels on Pd/ceria. *Appl. Catal. A.*, **224**, 209–218 (2002).

27. Ashihara, S., *Ensemble (in Japanese)*, ***11*(2)**, 20–24 (2009).

28. Eikerling, M., Kornyshev, A., Proton transfer in a single pore of a polymer electrolyte membrane. *J. Electroanal. Chem.*, **502**, 1–14 (2001).

29. Erdy-Gruz, T., Lengyel, S., Proton transfer in solution. *Modern Aspects Electrochem.*, **12**, 1–40 (1977).

30. Jones, G. *et al.* Low temperature water-gas shift: in situ DRIFTS-reaction study of ceria surface area on the evolution of formates on Pt/CeO_2 fuel processing catalysts for fuel cell applications. *Appl. Catal. A: Gen.*, **252**, 107–118 (2003).

31. Li, C., Xin, Q., FT-IR spectroscopic investigation of methane adsorption on cerium oxide. *J. Phys. Chem.*, **96**, 7714–7718 (1992).

33. Kerkeni, B., Clary, D. C., Quantum reactive scattering of H^+ hydrocarbon reactions. *Phys. Chem. Chem. Phys.*, **8**, 917–925 (2006).

36. Tuckerman, K., Laasonen, K., Sprik, M., Parrinello, M., *Ab initio* molecular dynamics simulation of the solvation and transport of H_3O^+ and OH^- ions in water. *J. Phys. Chem.*, **99(16)**, 5749–5752 (1995).

37. Priolkar, K. A. *et al.* Formation of $Ce_{1-x}Pd_xO_{2-\delta}$ solid solution in combustion-synthesized Pd/CeO_2 catalyst: XRD, XPS, and EXAFS investigation. *Chem. Mater.*, **14(5)**, 2120–2128 (2002).

38. Guo, X., Sigle, W., Maier, J., Blocking grain boundaries in yttria-doped and undoped ceria ceramics of high purity. *J. Am. Ceram. Soc.*, **86 (1)**, 77-87 (2003).

39. Jasinski, P., Petrovsky, V., Suzuki, T., Anderson, H. U., Impedance studies of diffusion

phenomena and ionic and electronic conductivity of cerium oxide, *J. Electrochem. Soc.*, **152 (4)**, J27-J32 (2005).

40. Tuller, H. L., Nowick, A. S., Small polaron electron transport in reduced CeO_2 single crystals, *J. Phys. Chem. Solids*, **38**, 859-867 (1977).

41. Trovarelli, A., Catalytic properties of ceria and CeO_2-containing materials, *Catal. Rev*., **38(4)**, 439-520 (1996).

42. Tredici, I. G., Maglia, F., Ferrara, V., Mustarelli, P., Tamburin, U. A., Mechanism of low-temperature protonic conductivity in bulk, high-density, nanometric titanium oxide, *Adv. Funct. Mater*. **24**, 5137–5146 (2014).

43. Liang, F. *et al.* Positive effect of water vapor on CO oxidation at low temperature over Pd/CeO_2-TiO_2 catalyst. *Catal. Lett*., **126**, 353–360 (2008).

44. Appel, L. G., Eon, J. G., Schmal, M., The CO_2-CeO_2 interaction and its role in the CeO_2 reactivity. *Catal. Lett*., **56**, 199–202 (1998).

45. Craciun, R., Daniell, W., Knozinger, H., The effect of CeO_2 structure on the activity of supported Pd catalysts used for methane steam reforming. *Appl. Catal. A*: *Gen*., **230**, 153–168 (2002).

46. Andrews, L., Wang, X., Alikhani, M. E., Manceron, L., Observed and calculated infrared spectra of $Pd(H_2)_{1,2,3}$ complexes and palladium hydrides in solid argon and neon. *J. Phys. Chem. A*., **105**, 3052–3063 (2001).

47. Shido, T., Iwasawa, Y., Regulation of reaction intermediate by reactant in the water-gas shift reaction on CeO_2, in relation to reactant-promoted mechanism. *J. Catal*., **136**, 493–503 (1992).

Supporting Figures

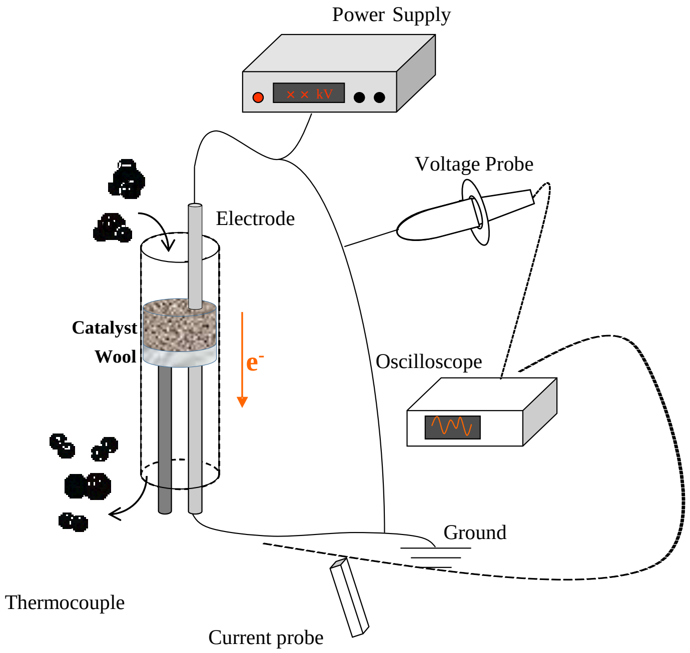

Figure S1 Schematic image of reactor.

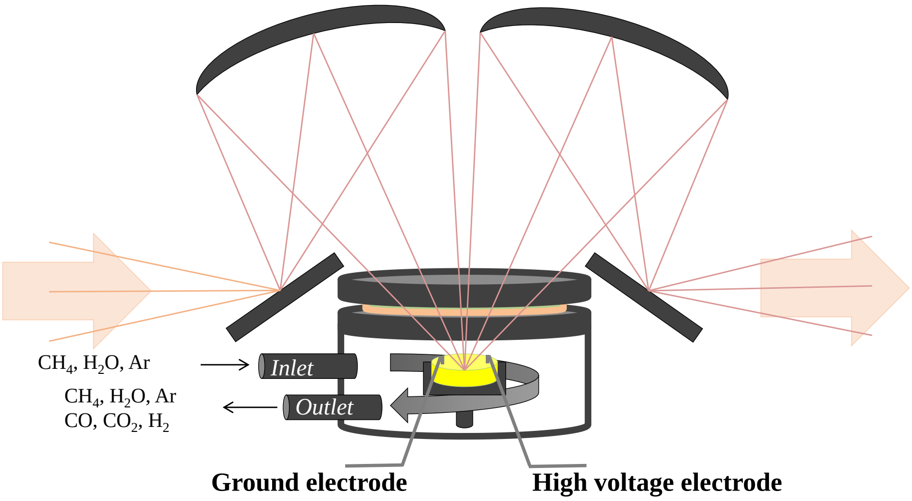

Figure S2 Schematic image of *operando*–DRIFTS measurement apparatus.

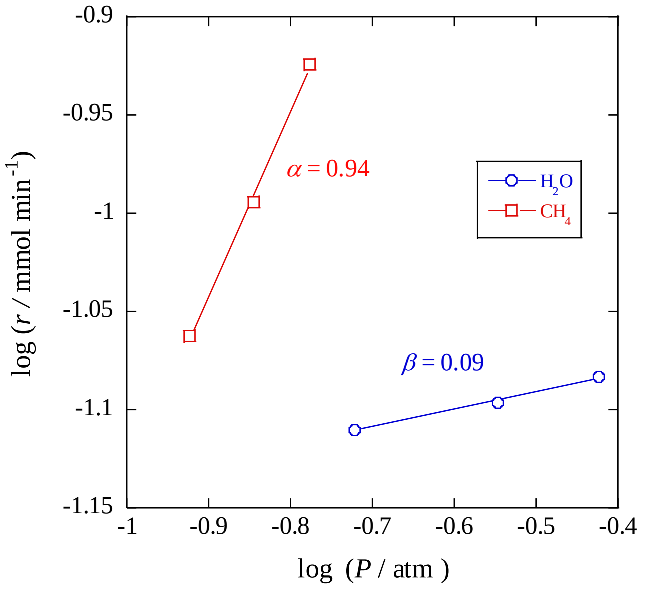

Figure S3 Methane and water pressure dependencies of reaction rate of SR in a kinetic range. Maximum methane conversion was 11.1%. Catalyst, 1.0wt%Pd/CeO_2 5 mg + SiO_2 500 mg; Flow, CH_4 : H_2O : Ar : He = 18 : 36 or 54 or 72 : 9 : balance and 22.5 or 27 or 31.5 : 72 : 9 : balance, total flow rate 180 SCCM; reaction temperature, 823 K.

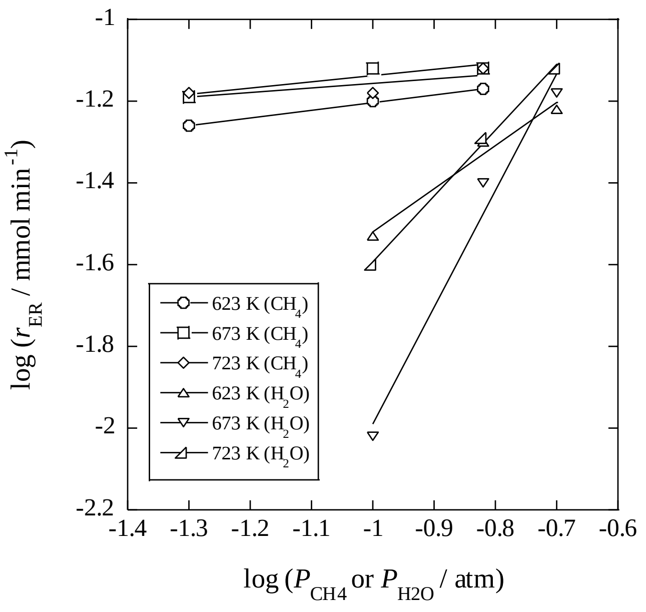

Figure S4 Methane and water pressure dependencies of r_{ER}. Dependence of methane pressure (α') was almost 0. Catalyst, 1.0wt%Pd/CeO_2 80 mg; flow, 120 SCCM (with 12 SCCM Ar as internal standard gas and He as balance gas); current, 5 mA.

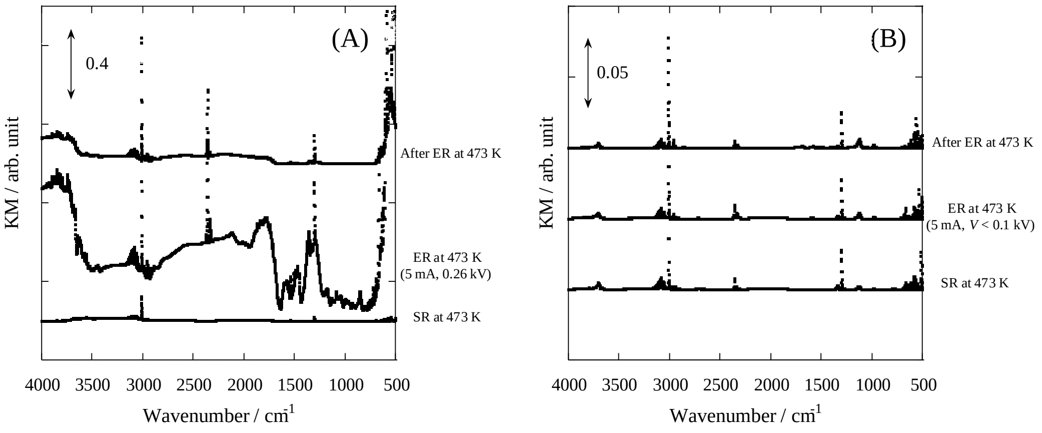

Figure S5 Raw data for *operando*-DRIFTS spectra with electric field. (A) With 1.0wt%Pd/CeO_2 catalyst, (B) with CeO_2 catalyst, temperature, 473 K; flow, CH_4 : H_2O : Ar = 1 : 2 : 62, total flow rate 65 SCCM; current, 0 or 5 mA.

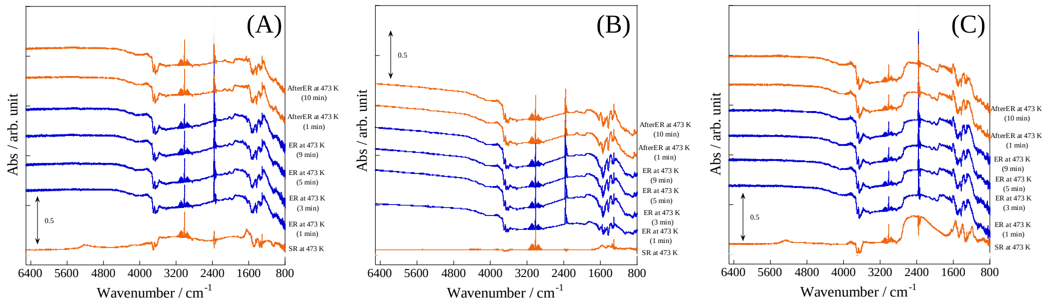

Figure S6 Raw data for *operando*-DRIFTS spectra with electric field. (A) With CH_4 and H_2O, (B) with only CH_4, (C) with CH_4 and D_2O, catalyst, 1wt%Pd/CeO_2; temperature, 473 K; flow, CH_4 : H_2O/D_2O : Ar = 1 : 2 or 0 : 62 or 64, total flow rate 65 SCCM; current, 0 or 5 mA.

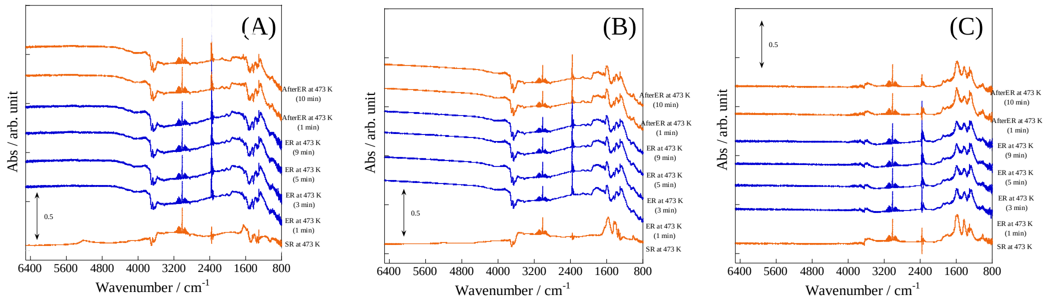

Figure S7 Raw data for *operando*-DRIFTS spectra with electric field. (A) At 473 K, (B) at 523 K, (C) at 573 K, catalyst, 1wt%Pd/CeO_2; flow, CH_4 : H_2O : Ar = 1 : 2 : 62, total flow rate 65 SCCM; current, 0 or 5 mA.

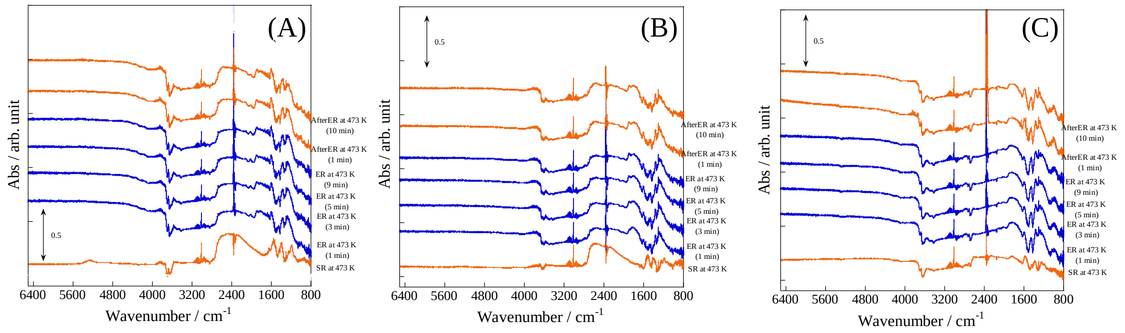

Figure S8 Raw data for *operando*-DRIFTS spectra with electric field. (A) At 473 K, (B) at 523 K, (C) at 573 K, catalyst, 1wt%Pd/CeO_2; flow, CH_4 : D_2O : Ar = 1 : 2 : 62 SCCM; current, 0 or 5 mA.

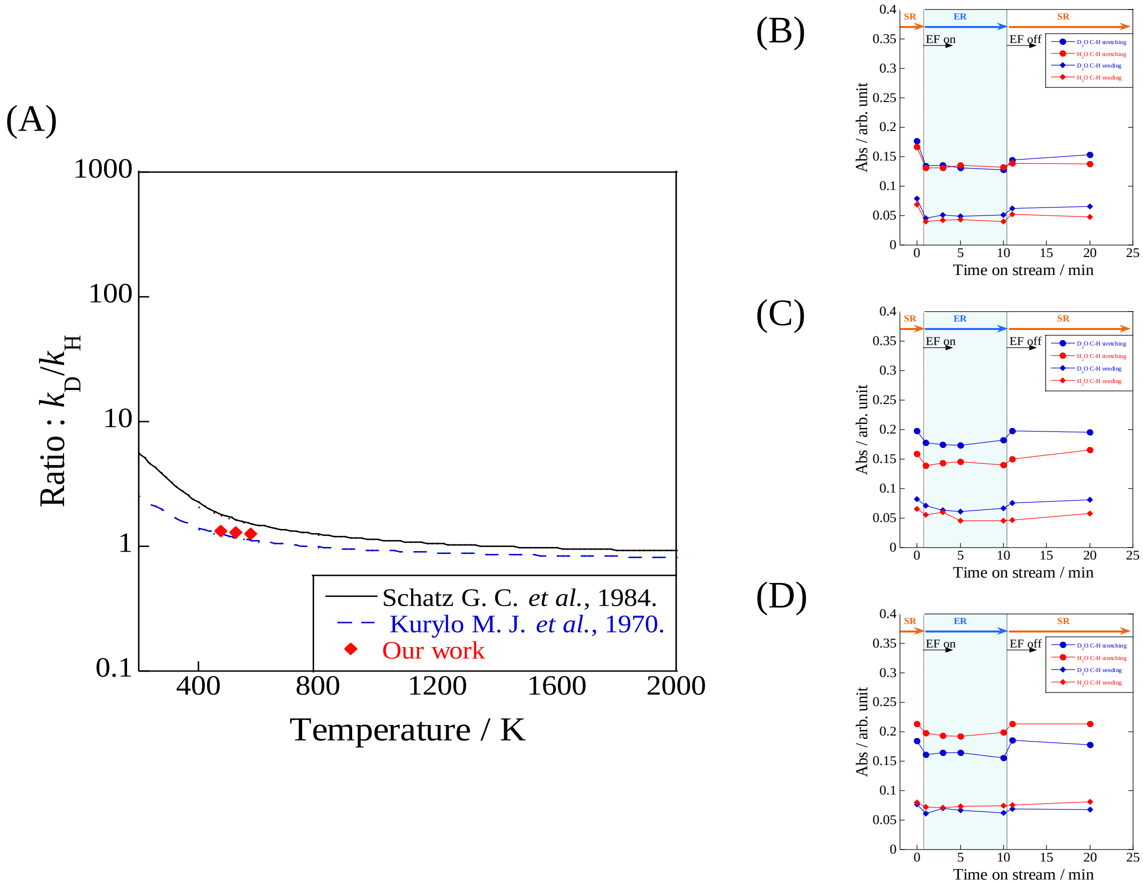

Figure S9 Inverse KIE. (A) Comparison between our work and reported experimental results and theoretical analysis, (B) *operando*-DRIFTS at 473 K, (C) *operando*-DRIFTS at 523 K, (D) *operando*-DRIFTS at 573 K, our work were within past reported trends as “ESR study of the kinetic isotope effect in the reaction of H and D atoms with CH_4” (*34*) and “A theoretical study of deuterium isotope effects in the reactions $H_2 + CH_3$ and H + CH_4” (*35*).

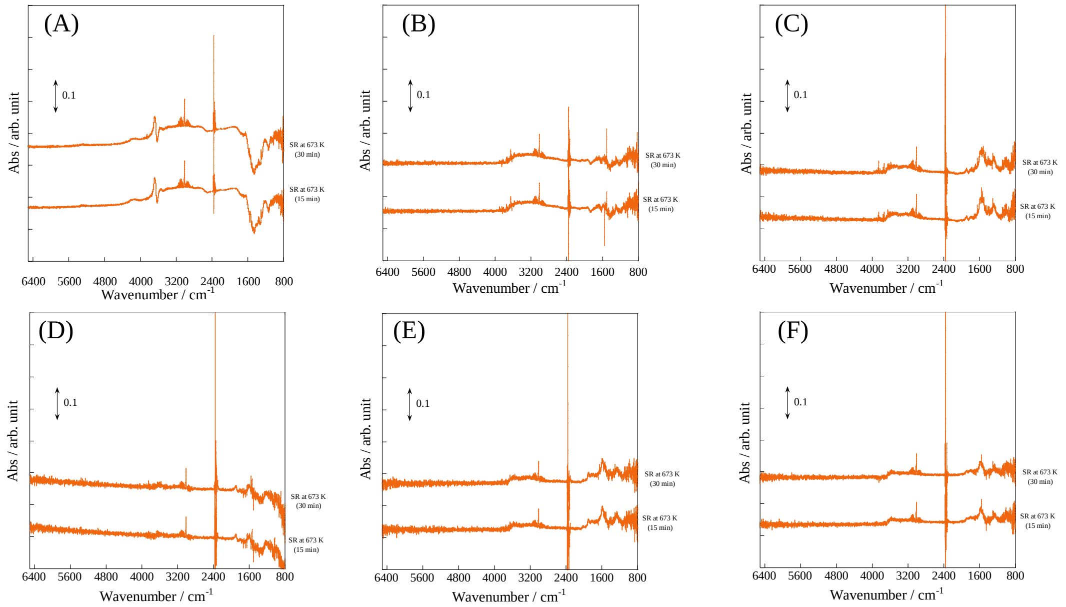

Figure S10 Raw data for *operando*-DRIFTS spectra of SR over various Pd catalyst. (A) CeO_2, (B) 0.5wt%Pd/CeO_2, (C) 1.0wt%Pd/CeO_2, (D) 2.0wt%Pd/CeO_2, (E) 3.0wt%Pd/CeO_2, (F) 5.0wt%Pd/CeO_2, temperature, 673 K; flow, CH_4 : H_2O : Ar = 1 : 2 : 62, total flow rate 65 SCCM.

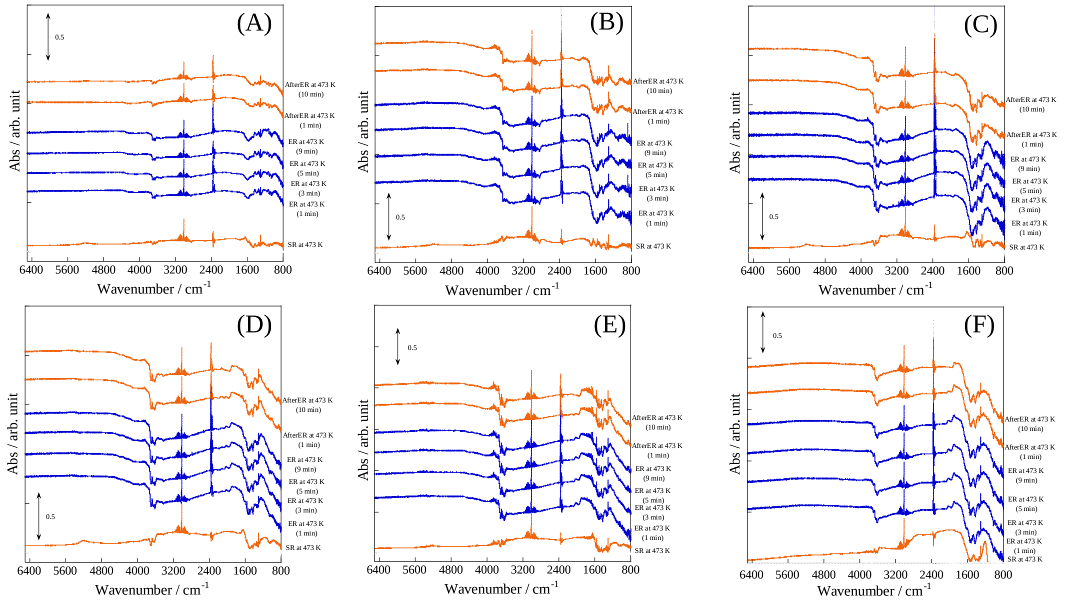

Figure S11 Raw data for *operando*-DRIFTS spectra of ER over various Pd catalysts. (A) CeO_2, (B) 0.5wt%Pd/CeO_2, (C) 1.0wt%Pd/CeO_2, (D) 2.0wt%Pd/CeO_2, (E) 3.0wt%Pd/CeO_2, (F) 5.0wt%Pd/CeO_2, temperature, 473 K; flow, CH_4 : H_2O : Ar = 1 : 2 : 62, total flow rate 65 SCCM; current, 5 mA.

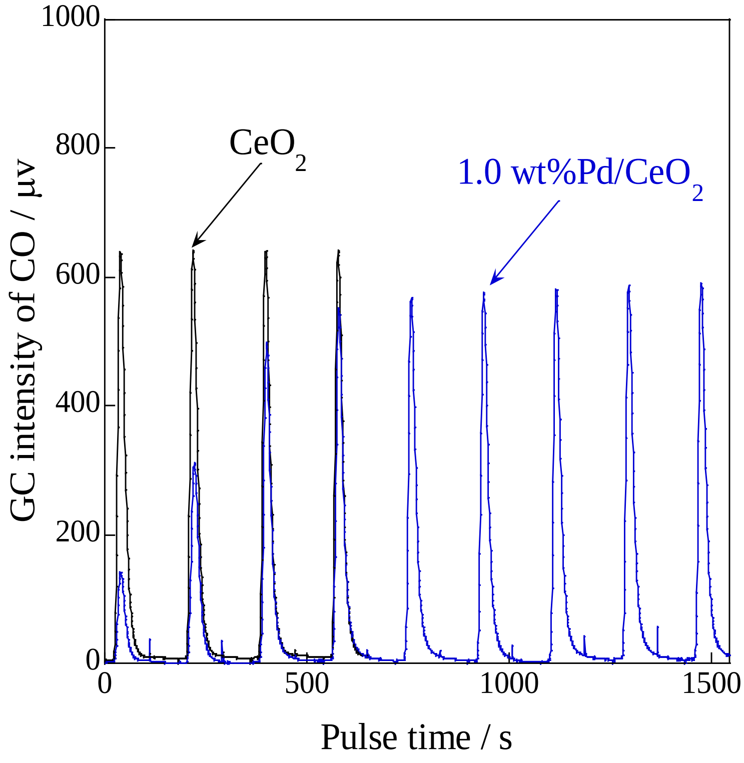

Figure S12 The results of CO pulse for CeO_2 and 1.0wt% Pd/CeO_2 catalyst at 323 K.

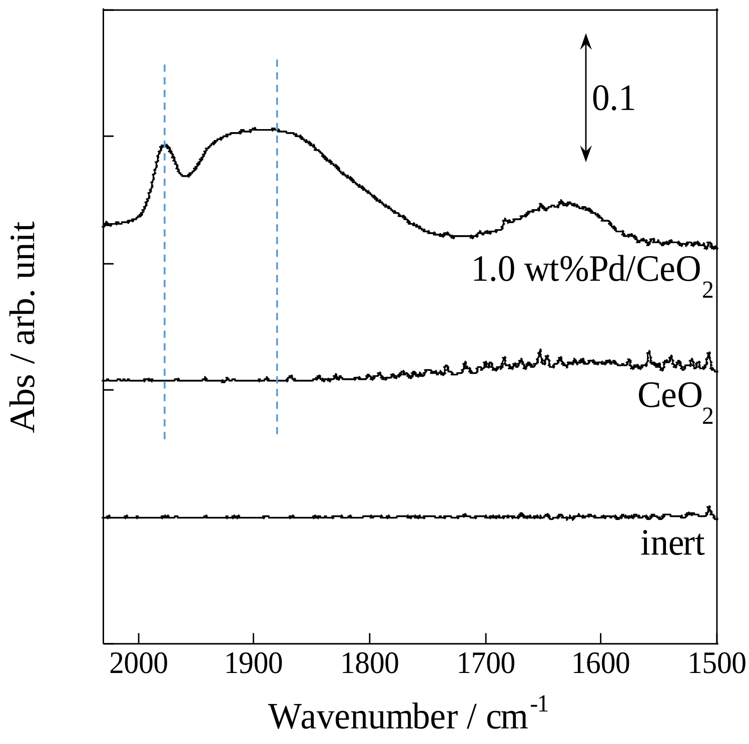

Figure S13 IR spectra recorded 1 h after supplying 40% CO flow with Ar at 323 K for each catalyst.

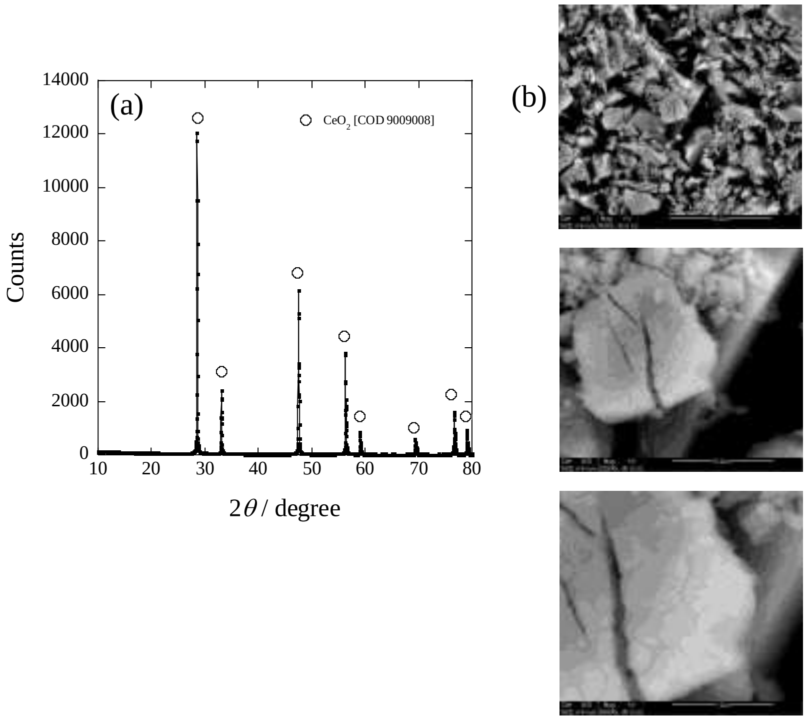

Figure S14 The results of characterization for CeO_2 disc. (a) XRD pattern (b) SEM images.

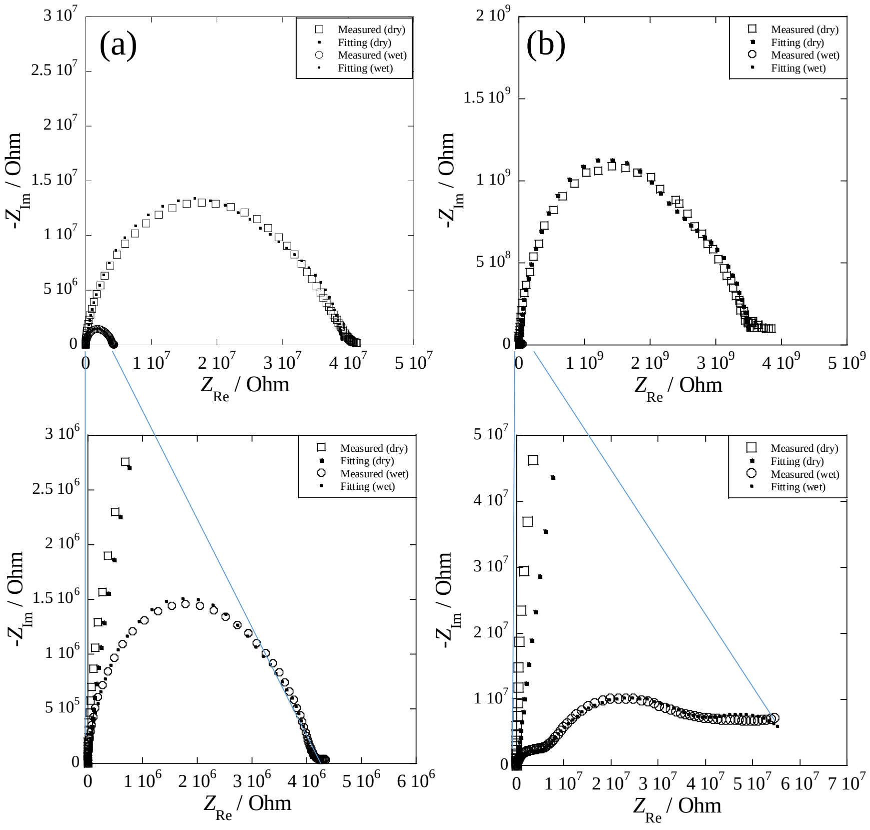

Figure S15 Nyquist plots for CeO_2 (a) at 673 K (b) at 473 K.

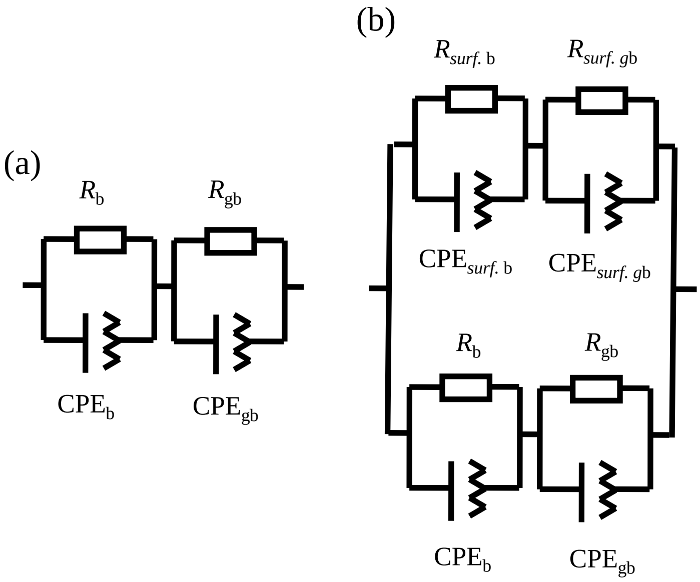

Figure S16 Equivalent circuit for analysis of impedance measurements (a) under dry condition (b) under wet condition; where *R* stands for the resistance, CPE represents the capacitance with constant phase element, and $_{b,\ gb,\ surf.\ b}$ and $_{surf.gb}$ respectively correspond to a bulk, a grain boundary, a surface of bulk, and a surface of grain boundary.

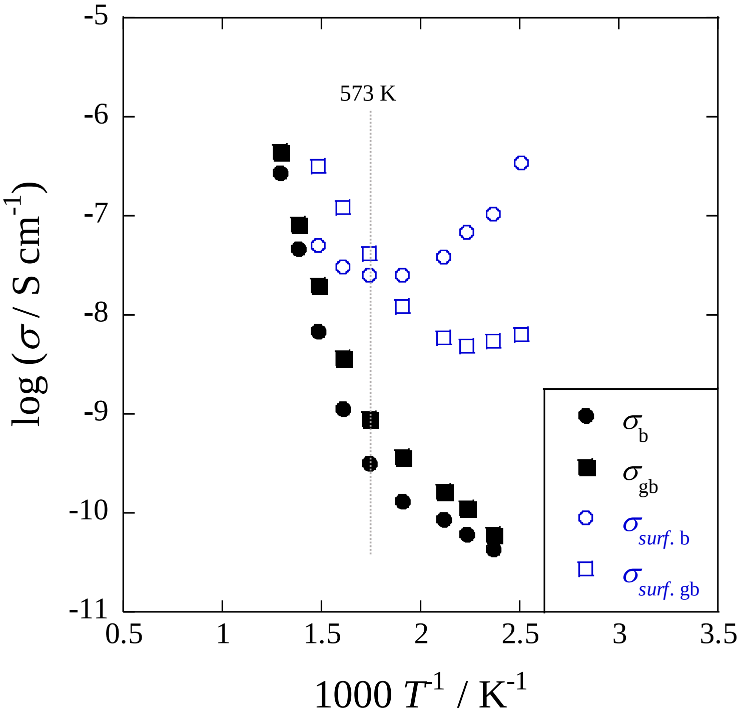

Figure S17 Temperature dependency of conductivity under dry and wet (P_{H2O} = 0.026 atm) conditions.

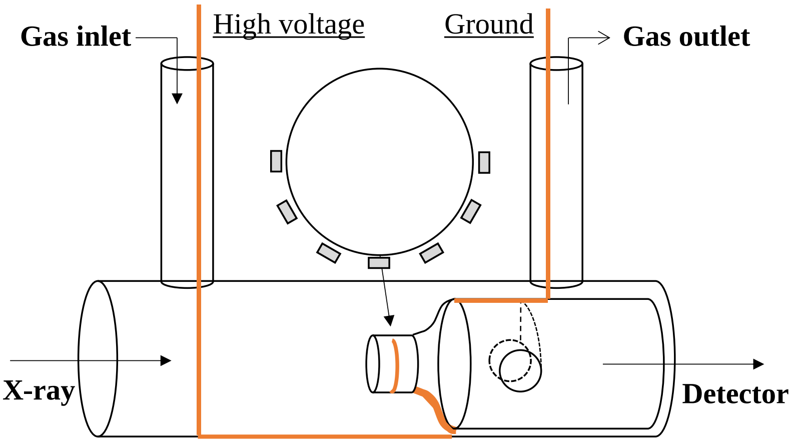

Figure S18 Schematic image of *in-situ* XAFS cell.

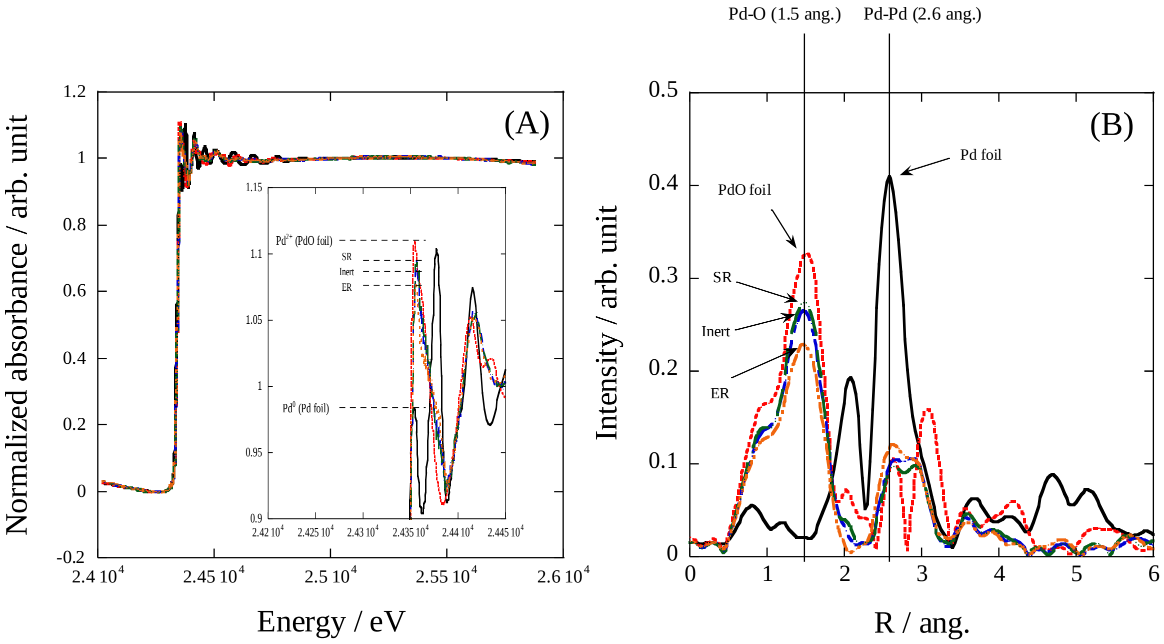

Figure S19 *In-situ* Pd-*K* edge XAFS measurements on 3wt%Pd/CeO_2 catalyst in the reaction condition with the electric field. (A) XANES spectra, (B) EXAFS spectra, gas flow, CH_4 : H_2O : Ar = 1 : 2 : 117, total flow rate 120 SCCM; temperature, 473 K; current, 5 mA.

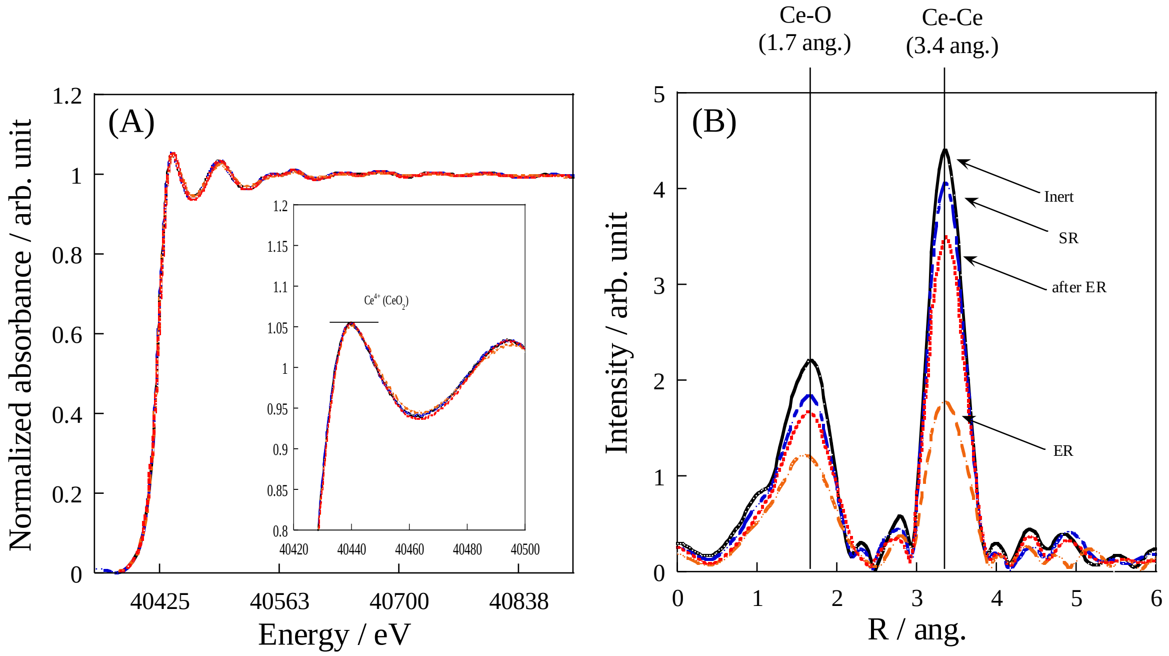

Figure S20 *In-situ* Ce-*K* edge XAFS measurements on 3wt%Pd/CeO_2 catalyst in the reaction condition with the electric field. (A) XANES spectra, (B) EXAFS spectra, gas flow, CH_4 : H_2O : Ar = 1 : 2 : 117, total flow rate 120 SCCM; temperature, 473 K; current, 5 mA.

Tables S1 Partial pressure dependence of reaction rates with/without electric field. Catalyst, 1.0wt%Pd/CeO_2, 80 mg; flow, 120 SCCM (with Ar as internal standard gas and He as balance gas); current, 0 or 5 mA.

T / K	Reaction SR or ER	P_{CH4} / atm	P_{H2O} / atm	*r* / mmol min^{-1}	*V* / kV	$r = k\,P_{CH4}^{\alpha}\,P_{H2O}^{\beta}$ α	β
473	ER	0.05	0.2	0.0316	0.25	0.32	0.79
		0.1		0.0417	0.21		
		0.15		0.0447	0.22		
		0.1	0.1	0.024	0.21		
			0.15	0.0309	0.21		
			0.2	0.0417	0.21		
623	SR	0.05	0.2	0.0102	–	0.32	0.31
		0.1		0.0132			
		0.15		0.0145			
		0.1	0.1	0.0120			
			0.15	0.0141			
			0.2	0.0148			
623	ER	0.05	0.2	0.0661	0.24	0.19	0.88
		0.1		0.0759	0.23		
		0.15		0.0813	0.23		
		0.1	0.1	0.0417	0.15		
			0.15	0.0646	0.21		
			0.2	0.0759	0.23		
673	SR	0.05	0.2	0.0282	–	0.23	0.25
		0.1		0.0324			
		0.15		0.0363			
		0.1	0.1	0.0269			
			0.15	0.0282			
			0.2	0.0324			
673	ER	0.05	0.2	0.0933	0.27	0.17	1.06
		0.1		0.110	0.26		
		0.15		0.112	0.22		
		0.1	0.1	0.0525	0.12		
			0.15	0.0794	0.18		
			0.2	0.110	0.26		
723	SR	0.05	0.2	0.0501	–	0.27	0.23
		0.1		0.0589			
		0.15		0.0676			
		0.1	0.1	0.0501			
			0.15	0.0550			
			0.2	0.0589			
723	ER	0.05	0.2	0.117	0.24	0.16	1.11
		0.1		0.126	0.18		
		0.15		0.141	0.19		
		0.1	0.1	0.0589	0.084		
			0.15	0.0955	0.14		
			0.2	0.126	0.18		

Tables S2 Kinetic analyses of methane steam reforming over various catalysts.

Catalyst	$r = k\, P_{CH4}^{\alpha}\, P_{H2O}^{\beta}$		*E*a	Reference
	α	β	kJ mol^{-1}	
Ir / ZrO_2	1	0	87	19
Ru / γ-Al_2O_3	1	0	91	20
Pt / ZrO_2	1	0	75	21
Rh / γ-Al_2O_3	1	0	109	22
Ni / MgO	1	0	102	23
Ni / $Ce_{0.75}Zr_{0.25}O_2$	1	-0.3	-	24
Ni / Al_2O_3	1	-0.3	-	24
Pd / ZrO_2	1	0	81	25
Pd / CeO_2	-	-	57	26
Pd / CeO_2 (With EF at 473 K)	0.32	0.79	14.3 (at lower temperature)	
Pd / CeO_2 (Without EF around 623 - 723 K)	0.27	0.26	54.4	Our work
Pd / CeO_2 (Without EF, at smaller amount of catalyst at 823 K)	0.94	0.09	-	

Tables S3 Kinetic analyses for SR and ER. Catalyst, 1.0wt%Pd/CeO_2, 80 mg; flow, CH_4 : H_2O : Ar : He = 12 : 24 : 12 : 72, total flow rate 120 SCCM; current, 0 or 5 mA.

preset *T*	$T_{thermocouple}$ SR	$T_{thermocouple}$ ER	Conversion SR	Conversion ER	Voltage	Reaction rate / mmol min^{-1}			Rate constant / mmol min^{-1}	
/ K	/ K	/ K	/ %	/ %	/ kV	r	r_{SR}	r_{ER}	k_{SR}	k_{ER}
398	398.2	441		5.9	0.21	0.029		0.029		0.091
423	424.1	456		5.0	0.21	0.032		0.032		0.11
448	450.0	480	0 (Detection Limit)	6.5	0.20	0.031	-	0.031	-	0.11
473	472.3	512		7.0	0.21	0.041		0.041		0.16
511	512.7	539		9.9	0.24	0.044		0.044		0.19
523	522.9	550	0.4	9.1	0.22	0.042	0.0015	0.041	0.0041	-
548	550.4	572	0.1	10.0	0.23	0.047	0.0041	0.043	0.012	0.195
573	574.1	605	1.0	11.1	0.23	0.051	0.0057	0.046	0.016	-
586	587.7	604	0.1	11.5	0.20	0.059	0.0079	0.051	0.022	0.25
623	622.3	646	2.6	15.5	0.23	0.076	0.013	0.062	0.037	0.34
648	649.6	669	4.0	15.9	0.23	0.085	0.020	0.065	0.056	0.56
673	671.9	697	7.3	21.8	0.26	0.11	0.032	0.077	0.092	1.0
723	716.9	731	13.6	24.9	0.18	0.13	0.059	0.066	0.17	6.5
773	774.6	784	22.5	33.5	0.16	0.16	0.11	0.049	0.31	35
823	821.7	828	31.9	38.1	0.13	0.18	0.15	0.029	0.43	154

Tables S4 Orders of water pressure at each reaction temperature for r_{ER}.

Reaction temperature	Order of water pressure
/ K	β'
473	0.83
623	1.05
673	1.61
723	2.85

Tables S5 Assignments for *operando*-DRIFTS spectra (as, asymmetry; s, symmetry).

Reaction SR or ER	Wavenumber / cm^{-1}	Species	Assignment	Reference
SR	3699, 3649	H_2O	O—H stretching	30
	2359	CO_2	O—C—O as	43
	1541	H_2O	O—H vending	-
	1429	Hydrogen carbonate		44
ER	3675, 3627	H_2O	O—H stretching	30
	3015	CH_4	C—H stretching	31
	2359	CO_2	O—C—O as stretching	43
	1793	CO or H	Pd_n—CO stretching or Pd—H stretching	45, 46
	1472	Mono Carbonate	O—C—O as stretching	47
	1361	Mono Carbonate	O—C—O s stretching	47
	1304	CH_4	C—H vending	31
	850	H_2O	O—H rotating	27

Tables S6 *Operando*-analyses using isotope. Catalyst, 1.0wt%Pd/CeO_2; flow, CH_4 : H_2O/D_2O : Ar = 1 : 2(0) : 62(64), total flow rate 65 SCCM; current, 5 mA; temperature, 473 K.

Reaction conditions	CH_4 conversion / %	Voltage / kV
CH_4 + H_2O	10.9	0.16
only CH_4	0.6	0.06
CH_4 + D_2O	11.5	0.17

Tables S7 *Operando*-analyses using isotope. Catalyst, 1.0wt%Pd/CeO_2; flow, CH_4 : H_2O/D_2O : Ar = 1 : 2 : 62, total flow rate 65 SCCM; current, 5 mA.

Temperature	H_2O				D_2O			
/ K	*V* / kV	CH_4 conversion / %	CO selectivity / %	CO_2 selectivity / %	*V* / kV	CH_4 conversion / %	CO selectivity / %	CO_2 selectivity / %
473	0.16	10.9	14.9	85.1	0.17	11.5	11.9	88.1
523	0.12	7.36	3.1	96.9	0.12	7.99	1.5	98.5
573	0.16	9.17	1.7	98.3	0.17	9.33	6	94

Tables S8 *Operando*-analyses over various catalysts. Flow, CH_4 : H_2O : Ar = 1 : 2 : 62, total flow rate 65 SCCM; current, 5 mA.

Pd / wt%	*Electreforming* (ER)					Catalytic reaction (SR)			
	T / K	*V* / kV	CH_4 Conversion / %	CO selectivity / %	CO_2 selectivity / %	*T* / K	CH_4 Conversion / %	CO selectivity / %	CO_2 selectivity / %
0	473	0.23	0.57	20.6	79.4	673	0.24	0	100
0.5		0.17	6.97	14.1	85.9		2.68	0	100
1		0.22	15	26.4	73.7		5	0	100
2		0.17	12.5	15.3	84.7		5.43	0	100
3		0.17	13.7	15.3	84.7		5.885	0	100
5		0.13	10.2	13.1	86.9		5.35	0	100

Tables S9 Results of CO pulse for various catalysts.

Pd / wt%	Pd Particle size / nm	Dispersion / %	Pd Surface Area / m^2 g^{-1}	Pd Perimeter / 10^9 m g^{-1}
0	-	-	-	-
0.5	1.21	91.9	2.05	3.38
1	1.15	96.6	4.32	7.48
2	1.83	60.9	5.44	5.94
3	2.13	52.5	7.05	6.63
5	3.14	35.6	7.96	5.07
